# Comprehensive pathological insights and molecular detection of caseous lymphadenitis caused by *Corynebacterium pseudotuberculosis* in slaughtered sheep, goats, and cattle

**DOI:** 10.5455/javar.2026.m1037

**Published:** 2026-03-31

**Authors:** Nazneen Sultana, Mohammad Abu Hadi Noor Ali Khan, Munmun Pervin, Sajeda Sultana, Moutuza Mostaree, Anika Tabassum, Mahmuda Islam

**Affiliations:** 1 Department of Pathology, Faculty of Veterinary Science, Bangladesh Agricultural University (BAU), Mymensingh 2202, Bangladesh; 2 Department of Pathology, Faculty of Animal Science and Veterinary Medicine, Sher-e-Bangla Agricultural University, Sher-e-Bangla Nagar, Dhaka 1207, Bangladesh

**Keywords:** *Corynebacterium pseudotuberculosis*, gross pathology, histopathology, PCR, ruminants

## Abstract

**Objectives:** Caseous lymphadenitis (CLA) is one of the significant economically important diseases of small ruminants caused by *Corynebacterium pseudotuberculosis*. Despite the occurrence of CLA worldwide, there are no reports of CLA in slaughtered ruminants in Bangladesh. This study was designed to investigate the pathology and molecular detection of CLA in slaughtered sheep, goats, and cattle in Bangladesh.

**Materials and Methods:** A total of 102 goats, 16 sheep, and 50 cattle were examined at several slaughterhouses in Mymensingh Sadar. Mesenteric lymph nodes, lungs, liver, spleen, and kidneys from animals with suspected internal lesions were evaluated pathologically. Confirmation of *C. pseudotuberculosis* was performed by polymerase chain reaction (PCR).

**Results:** Gross examination revealed enlarged mesenteric lymph nodes with multiple abscesses containing greenish-yellow caseous material. The spleen exhibited a characteristic onion-skin appearance, commonly associated with CLA. Histopathology revealed caseous necrosis, encapsulated by fibrous tissue, with infiltration of neutrophils and mononuclear cells, along with Gram-positive coccobacilli arranged in Chinese letter patterns. Similar lesions were observed in other visceral organs of goats. PCR detected *C. pseudotuberculosis* in 29.41% (30/102) goats, 6.25% (1/16) sheep, and 18% (9/50) cattle.

**Conclusions:** To date, it is the first report of internal CLA in slaughtered ruminants in Bangladesh, as confirmed by pathological and molecular findings, and it highlights the importance of slaughterhouse-based surveillance for disease control.

## 1. Introduction

Caseous lymphadenitis (CLA) is a chronic, often subclinical bacterial disease of small ruminants caused by *Corynebacterium pseudotuberculosis*, a Gram-positive facultative intracellular coccobacillus [1]. The pathogen affects a wide range of animal species, including cattle and humans [2, 3]. In small ruminants, infection occurs mainly through contact with purulent abscesses, secretions, or contaminated materials or via skin wounds, ingestion, or inhalation [4, 5, 6]. After entry, bacteria spread from local lymph nodes to visceral organs such as the lungs, liver, and spleen, facilitated by phospholipase D (PLD), which enhances vascular permeability and tissue invasion [7, 8]. Accordingly, CLA manifests as superficial or internal forms in sheep and goats, whereas in cattle, it may present as cutaneous, mastitic, or internal disease [9, 10].

The disease causes substantial economic losses in the livestock industry, particularly in small ruminants, through reduced productivity, including weight loss, decreased meat and milk yield, impaired fertility, and carcass and organ condemnation at slaughter, especially in Asia, Africa, and Central and South America. Despite its economic significance, the true prevalence of CLA remains poorly understood, as the disease is often neglected due to subclinical infections and underdiagnosis at slaughter [11]. Reported prevalence varies widely across countries (8–90%) [12]. In Bangladesh, superficial lymphadenitis has been reported in goats in the Mymensingh and Kushtia districts, with prevalences of 6.59% and 0.74%, respectively [13, 14]; however, to date, no reports have been published on the detection of internal CLA in slaughtered ruminants. Post-mortem evaluation at slaughterhouses provides a critical opportunity to detect characteristic internal lesions in lymph nodes and visceral organs that would otherwise remain unrecognized [15]. Assessment of these lesions allows evaluation of disease distribution, severity, and prevalence within slaughter populations [4]. However, the gross and histopathological features of CLA can overlap with those of other chronic infectious conditions; pathological findings alone are not sufficient for definitive diagnosis [4, 16].

Diagnosis of *C. pseudotuberculosis* traditionally relies on clinical lesion assessment and phenotypic and biochemical tests [17, 18], while serological assays are mainly used for herd-level antibody detection [14]. Nowadays, PCR-based methods targeting the *16S rRNA* [17], *pld* [19], and *rpoB* [20] genes have been developed due to their high sensitivity and diagnostic efficiency.

Previously, *C. pseudotuberculosis* DNA had been successfully detected in sheep and goats by PCR [17, 21]. However, molecular data from slaughtered sheep, goats, and cattle in Bangladesh remain limited. Molecular identification of *C. pseudotuberculosis* improves diagnostic accuracy, and integrating pathological assessment with molecular confirmation in slaughtered animals improves disease surveillance, meat inspection, and epidemiological control of CLA.

Therefore, this study aimed to investigate the pathological insights and molecular detection of the internal form of CLA in slaughtered sheep, goats, and cattle in Bangladesh, emphasizing the need for routine slaughterhouse-based surveillance and effective control strategies.

## 2. Materials and Methods

### 2.1. Ethical approval

The Ethical Standard of Study Committee, Bangladesh Agricultural University, approved the research project on May 11, 2019, under reference number BAURES/ESRC/VET/05.

### 2.2. Sample collection and gross pathological assessment

A total of 168 animals (goats = 102, sheep = 16, and cattle = 50) were screened from seven slaughterhouses in the Mymensingh division, including K.R. Market, Shesmor, Bolashpur, Kewatkhali, Pauro Super Market, Ganginar Par, and Mechua Bazar between July 2019 and January 2022. The sample size was determined by animal availability at the slaughterhouses rather than by a priori statistical calculation. Goats were slaughtered more frequently than cattle and sheep, resulting in a higher sample size for this species. The slaughtered animals included Black Bengal goats, local indigenous cattle, and indigenous sheep, all of which appeared clinically healthy at the time of slaughter. However, detailed information on age and sex could not be obtained because of inadequate recordkeeping and management practices at the slaughterhouses. During post-mortem examination, the lymph nodes and visceral organs (lungs, liver, spleen, and kidneys) were carefully inspected for any gross lesions indicative of CLA infection. The gross changes observed were recorded at slaughter.

Enlarged and inflamed mesenteric lymph nodes and suspected visceral organs were collected in sterile Falcon tubes and delivered to the lab for further histopathological, impression smear staining, and molecular detection of CLA by PCR. Small parts of the tissue samples from suspected animals, along with their healthy parts, were incised with a sharp blade and fixed in 10% neutral buffered formalin (NBF) for histopathology [22]. Small pieces of the tissue samples were also aseptically incised, collected in Eppendorf tubes, cryopreserved, and stored at –20°C. Genomic DNA from the frozen samples was extracted and used in PCR to detect the causal agent of caseous lymphadenitis.

### 2.3. Impression smear staining

Impression smears were taken aseptically from the cut surface of the edges of the caseous materials of suspected lymph nodes, liver, lungs, kidney, and spleen. The produced smears were air-dried, fixed by transient heating, stained with Gram’s stain [22], and examined under a high-power (100x) microscope. Microphotographic techniques (Cell Bioscience, Alphaimager HP, California, USA) were used to capture images according to the manufacturer’s guidelines [23].

### 2.4. Staining of tissue sections with hematoxylin and eosin (H & E) and Goldner’s trichrome

Representative tissue samples were processed using standard procedures and stained with hematoxylin and eosin (H & E) [22]. Also, Goldner’s trichrome staining [24] was performed to characterize cellular reactions and responses. A microphotography system (Cell Bioscience, Alphaimager HP, California, USA) was used to capture tissue changes, and the images were subsequently analyzed and documented according to the manufacturer’s guidelines [23].

### 2.5. Bacterial identification by Gram staining in tissue sections

Tissue sections were subjected to Gram staining to identify bacterial type (Gram-positive or Gram-negative) in the lungs, liver, spleen, kidneys, and lymph nodes, according to standard procedures [22]. Gram-positive bacteria showed a purple color; in contrast, Gram-negative bacteria showed a pink color under a microscope at high magnification (100x).

### 2.6. Genomic DNA extraction from tissues

Microbial DNA was extracted from suspected samples for PCR detection of *C. pseudotuberculosis* according to the instructions for the Wizard Genomic DNA Purification Kit (Promega, USA) [25]. In brief, tissues were homogenized and lysed using heat incubation in genomic lysis solution. RNA contamination was then eliminated by RNase treatment. Cellular proteins were removed by a salt-based precipitation step followed by centrifugation. DNA was then precipitated with isopropanol, washed with 70% ethanol, air-dried, and dissolved in nuclease-free water. The Nanodrop™ spectrophotometer (IAEA, Seibersdorf, Vienna) was used to measure DNA concentration and purity at 260 nm/280 nm. The isolated DNA was kept at –20°C for subsequent analysis.

### 2.7. Molecular detection of C. pseudotuberculosis by PCR

To confirm *C. pseudotuberculosis*, a uniplex PCR targeting the *16S rRNA* gene was performed. The *16S rRNA* gene was selected for initial screening due to its high specificity for molecular identification of *C. pseudotuberculosis*. However, as *16S rRNA* cannot reliably differentiate *C. pseudotuberculosis* from *C. ulcerans*, the virulence-associated *pld* gene [19] and the species-specific *rpoB* gene [20] were further targeted using duplex PCR to enhance diagnostic specificity and ensure accurate confirmation of pathogenic *C. pseudotuberculosis*. Primers targeting the *16S rRNA* gene were designed by downloading nucleotide sequences from the NCBI web database (GeneBank accession X84255.1), and primers targeting the *pld* [19] and *rpoB* [20] genes of *C. pseudotuberculosis* were obtained from earlier research. Table 1 lists the oligonucleotide primers used in this investigation [19, 20]. One Taq ^R^ Quick-Load ^R^2X Master Mix kit (New England Biolabs, USA) was used for PCR, with a reaction volume of 25 μl. Briefly, the PCR reaction mixture was prepared with a 2x PCR master mix provided by the kit, incorporating 20 pmol of each primer (for duplex PCR, both primer sets were included). A total of 150–200 ng of DNA template was added, and nuclease-free water was used to adjust the final volume to 25 µl. A negative control was prepared by substituting the DNA template with nuclease-free water H₂O to assess for contamination, while a positive control with known target DNA was included to confirm proper amplification. The reaction was conducted using a Proplex Gradient PCR, a USA oil-free thermal cycler.

**Table 1.** Oligonucleotide primers used in PCR detection of *Corynebacterium pseudotuberculosis* in slaughtered sheep, goats, and cattle.

Genes Targeted	Primers name	Primer sequence (5′–3′)	Amplicon size (bp)	Reference
*16S rRNA*	CorPsuF1	CGA ACG GGT GAG TAA CAC GTG	632	Accession No. (X84255.1) This work
	CorPsuR1	CGT CAG TTA CTG CCC AGA GAC		
*pld*	PLD-F	ATA AGC GTA AGC AGG GAG CA	203	[19]
	PLD-R2	ATC AGC GGT GAT TGT CTT CCA GG		
*rpoB*	C2700F	CGT ATG AAC ATC GGC CAG GT	446	[20]
	C3130R	TCC ATT TCG CCG AAG CGC TG		

The thermal profile of PCR amplification included 35 cycles, with the initial denaturation taking place at 94°C for 3 min, followed by denaturation at 94°C for 30 sec, annealing at 61°C for 1 min (*16S rRNA* gene) and 58°C for 40 sec (*pld* and *rpoB* genes) [19, 20], extension at 68°C for 5 min, and final extension taking place at 68°C for 7 min.

PCR amplicons were electrophoresed using a WSE-1710 Submerge-Mini2322100 apparatus (China). After that, a transilluminator (Alpha imager, USA) was used to capture the photos. The amplicon sizes in the agarose gel were determined with a 100 bp DNA ladder (TackIT, Invitrogen, USA).

## 3. Results

This study has limitations due to the unequal and limited sample sizes, particularly for sheep; therefore, no statistical comparisons among species were performed, and the results are presented descriptively. The summary of gross pathological lesions and PCR detection of *C. pseudotuberculosis* in slaughtered ruminants is summarized in Table 2.

**Table 2.** Gross pathological lesions and PCR detection of *Corynebacterium pseudotuberculosis* in slaughtered ruminants.

Organ Involvements	Species			Gross pathological findings	PCR positive (n, %)
	Goats (n = 102)	Sheep (n = 16)	Cattle (n = 50)		
Mesenteric lymph nodes	28	1	3	Markedly enlarged lymph nodes ranging from 2.5 to 8 cm in size with multiple abscesses containing greenish–yellow, cheesy caseous material in goats, sheep, and cattle; calcification in approximately half of the goats	Goats: 30 (29.41%)Sheep: 1 (6.25%)Cattle: 9 (18%)
Liver	8	1	0	Variable-sized nodules with brownish to yellowish caseous material on the cut surface	Included above
Spleen	3	0	0	Thick fibrous capsule; concentric alternating layers of caseous and friable material (onion-skin appearance)	Included above
Lungs	3	0	1	Suppurative bronchopneumonia; multiple abscesses in the lung parenchyma and greenish purulent exudate on sectioning of 2 goats and one cattle.Small pears to the walnut size of multiple abscesses (discrete nodules) were also recorded in the lung parenchyma of one goat.	Included above
Kidneys	1	0	0	Moderately sized nodules with brownish caseous material	Included above
Superficial lesions	0	0	0	No superficial abscesses or wounds observed	Included above

### 3.1. Gross pathological observations

**Figure 1.**
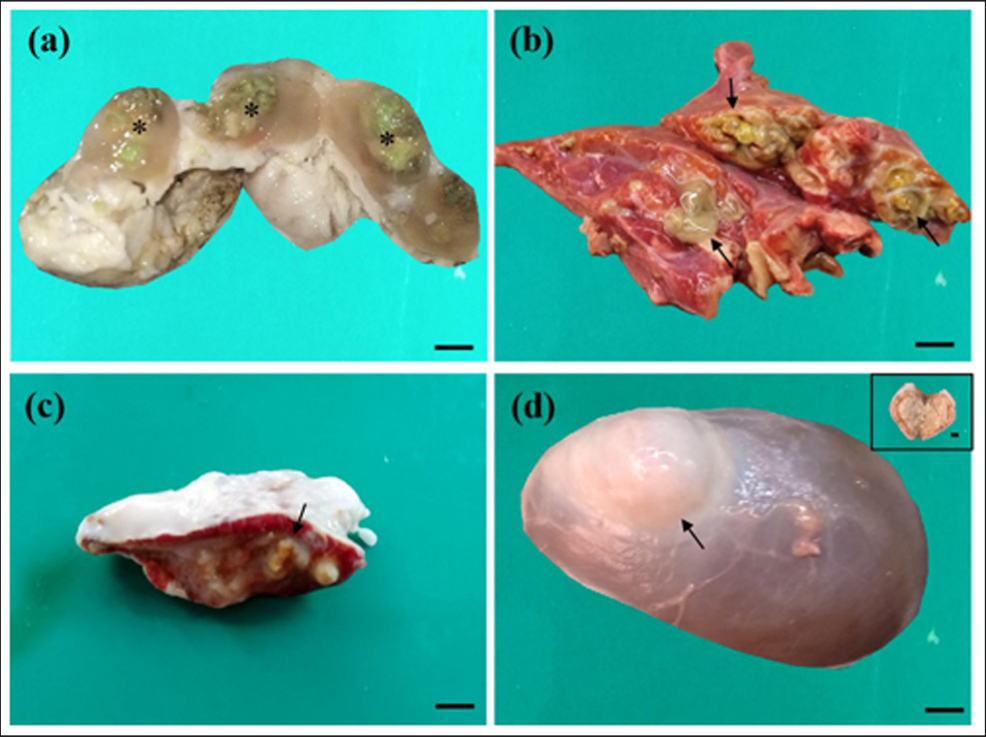
Gross pathological findings of the mesenteric lymph node, spleen, and kidney of a caseous lymphadenitis-affected goat (a, c–d) and the lungs of cattle (b). Abscesses filled with greenish, caseous, purulent material (asterisks) were observed after cutting the mesenteric lymph node (a). Thick, creamy pus (arrows) oozed out from abscesses at the lung’s cut surface (b). A semisolid caseous mass grew as an onion skin pattern (arrow) was observed after excision of the spleen (c). A large tubercle (arrow) filled with yellowish, caseous, purulent material (inset) was revealed in the kidney (d). Bar = 2 cm.

Among 168 animals (goats = 102, sheep = 16, and cattle = 50) investigated at slaughter, superficial lymphadenitis, abscesses, or wounds were not observed in any samples. Interestingly, moderate-to-severe lymphadenitis measuring 2.5–8 cm in diameter was observed in the animals’ mesenteric lymph nodes. Gross examination revealed marked enlargement of the lymph nodes in 28 goats, 1 sheep, and 3 cattle, with multiple abscesses containing greenish, cheesy, caseous material (Figure 1a). Calcified granules were also recorded in approximately half of the goats. Variable-sized nodules were observed in the livers of 8 goats and 1 sheep. After cross-sectioning the nodule, brownish to yellowish caseous material was revealed. The spleen of 3 goats was covered with a thick fibrous capsule. The cut surfaces of the spleen showed thick, semi-solid fluid with brownish to yellowish concentrated pus and a concentrated fibrous connective tissue layer with alternating zones of caseous and friable material, giving the characteristic onion skin appearance (pathognomonic lesions of CLA) (Figure 1c). In the lungs, suppurative bronchopneumonia was observed in 2 goats and 1 cattle, a characteristic lesion for *C. pseudotuberculosis* infection. In addition, small peas to the walnut size of multiple abscesses (discrete nodules) were also recorded in the lung parenchyma of 1 goat. The affected lungs were red to gray, solid, and pale. Suppurative exudates (greenish pus) oozed out following sectioning of the affected lobes of the lungs (Figure 1b). The kidneys were the least commonly affected organs in this study. Moderately larger nodules were observed in the kidneys of 1 goat, and, after the cross-section of the nodule, brownish caseous materials were seen (Figure 1d). In all cases, the characteristic purulent material was non-odorous. None of the cattle and sheep showed visible lesions in the spleen and kidney during this study.

### 3.2. Impression smear examination

**Figure 2.**
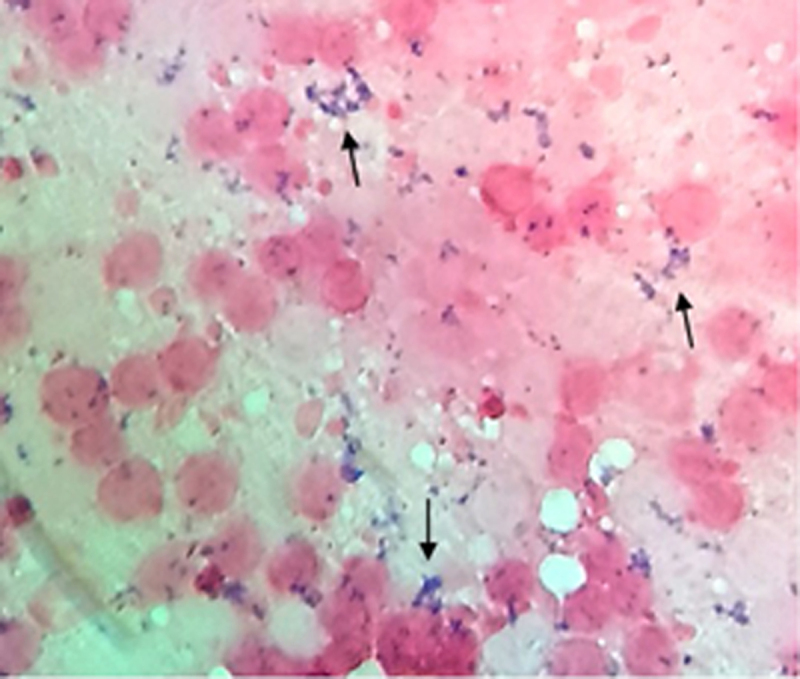
Impression smear staining of the mesenteric lymph node of a caseous lymphadenitis-affected goat. Pleomorphic blue-colored gram-positive coccobacilli arranged in a Chinese pattern (arrows) characteristic of *Corynebacterium pseudotuberculosis* were seen. Gram stain. Bar = 50 µm.

The Gram stain of impression smears from lymph nodes and visceral organs revealed pleomorphic, blue-colored Gram-positive coccobacilli arranged in a Chinese pattern, characteristic of *C. pseudotuberculosis* in the tissue samples (Figure 2).

### 3.3. Observation of tissue sections stained with hematoxylin and eosin (H & E)

**Figure 3.**
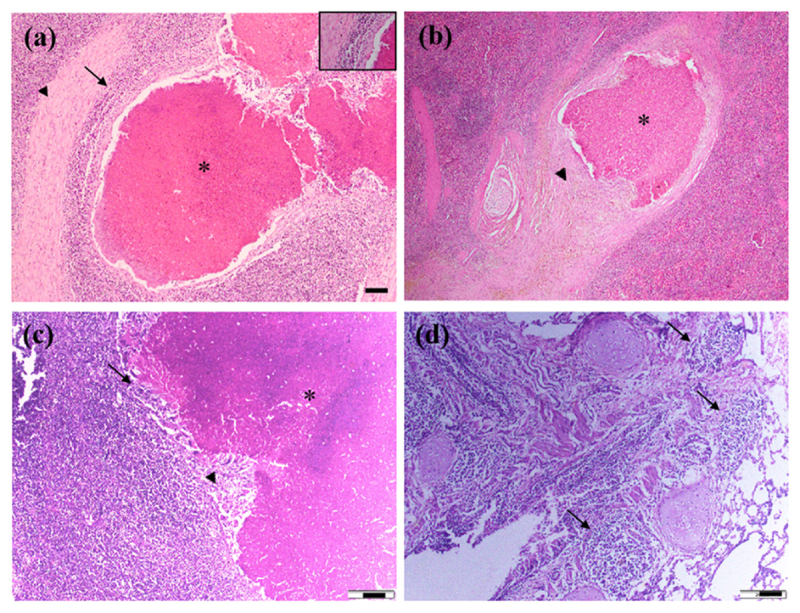
Histopathological findings of the mesenteric lymph node and spleen of goats (a–b) and the mesenteric lymph node and lung of cattle (c–d). A central caseous necrotic core (asterisk) was seen, surrounded by a thick fibrous capsule (arrowhead) and inflammatory cells (arrow); neutrophils, lymphocytes, and macrophages (inset: higher magnification) were predominantly infiltrated (a). Lesions were similar in the spleen of goats and the mesenteric lymph node of cattle (b–c). Huge infiltration of inflammatory cells was also seen in the lung parenchyma (arrow) (d). H & E stain; bar (a, inset a, and c–d are 100 µm; b is 200 µm).

Mesenteric lymph nodes of goats, sheep, and cattle revealed lesions with a central caseous necrotic core, with or without calcification, surrounded by proliferating fibrous connective tissue (Figures 3a, 3c). The caseo-necrotic core outside the membrane was surrounded by inflammatory cells, predominantly neutrophils, lymphocytes, and macrophages (Figure 3a). The lungs (Figure 3d), liver, spleen (Figure 3b), and kidneys of affected tissues also showed similar lesions.

### 3.4. Bacterial identification by Gram staining in tissue sections

**Figure 4.**
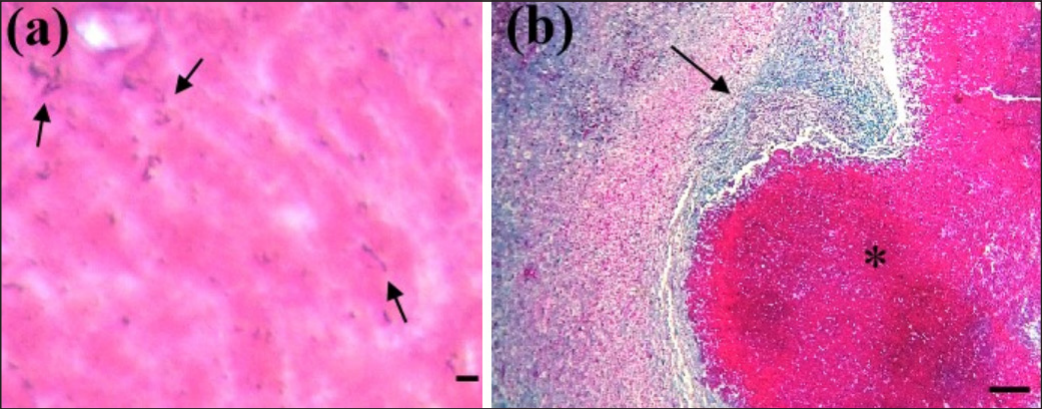
Histopathological findings of the mesenteric lymph node of a goat. The Chinese letter shape arrangement of gram-positive coccobacilli (arrows) was seen in the caseous center of the mesenteric lymph node (a). A central caseous necrotic core was seen (asterisk), surrounded by a blue-colored thick fibrous capsule (arrow) with infiltration of inflammatory cells (b). Gram’s staining (a); Goldner’s trichome staining (b). Bar (a is 50 µm; b is 100 µm).

Gram’s staining of tissue sections from lymph nodes and visceral organs revealed Gram-positive coccobacilli in the caseous necrotic zone, which were arranged in the shape of a Chinese letter (Figure 4a).

### 3.5. Goldner’s trichome staining of tissue sections

Suspected tissue sections of mesenteric lymph nodes, liver, kidney, and spleen were also stained by Goldner’s trichrome for distinct visualization of connective tissues. Proliferation of blue-colored fibrous connective tissue was clearly visible in and around the infected tissues in our investigation (Figure 4b).

### 3.6. Results of PCR

**Figure 5.**
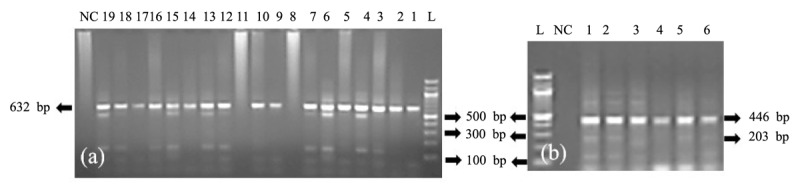
Uniplex PCR results of *the Corynebacterium pseudotuberculosis 16S rRNA* gene, which includes 632 bp segments from goats, sheep, and cattle. L = DNA marker for 100 bp, NC = negative control, 1–5 = representative goat mesenteric lymph node isolates, 6–7 = representative goat liver isolates, 9 = representative goat spleen isolate, 10 = representative goat kidney isolate, 12 = representative sheep liver isolate, and 13–18 = representative cattle mesenteric lymph node isolates; 19 = representative cattle lung isolate (a). The duplex PCR results for the *C. pseudotuberculosis pld* and *rpoB* genes, including 203 bp and 446 bp segments from goats, sheep, and cattle. L = DNA marker for 100 bp, NC = negative control, 1–2 = representative goat mesenteric lymph node isolates; 3 = representative goat liver isolate; 4 = representative goat spleen isolate; 5 = representative sheep liver isolate; 6 = representative cattle mesenteric lymph node isolate (b).

In this study, 168 animals were examined, and *C. pseudotuberculosis* was detected by PCR. Uniplex PCR detected a 632 bp fragment of the *16S rRNA* gene (Figure 5a) of *C. pseudotuberculosis* in 29.41% (30/102) of goats, 6.25% (1/16) of sheep, and 18% (9/50) of cattle. All positive samples were retested using duplex PCR with a different set of primers targeting the *pld* and *rpoB* genes of *C. pseudotuberculosis*. PCR amplification products of expected size, i.e., 203 bp and 446 bp (Figure 5b), respectively, were obtained for the *pld* and *rpoB* genes of pathogenic *C. pseudotuberculosis* in all 30 goats, 1 sheep, and 9 cattle.

## 4. Discussion

The present study demonstrates the occurrence of CLA in slaughtered sheep, goats, and cattle in Bangladesh. Slaughterhouse-based surveillance plays a crucial role in infectious disease control and eradication programs worldwide by providing reliable data on the prevalence of economically important animal diseases, including zoonoses [26, 27]. The absence of superficial lymphadenitis, abscesses, or wounds during ante-mortem inspection, together with the detection of characteristic internal lesions at post-mortem examination, indicates the disease’s subclinical form [1] and highlights the importance of slaughterhouse surveillance for identifying CLA. The subclinical form of CLA showed internal lesions in mesenteric lymph nodes and various visceral organs without any evidence of the production of external lesions. In small ruminants, CLA mainly occurs in two forms: abscesses that occur in the superficial lymph nodes are indicative of the disease’s external form, and abscesses that form in the lymph nodes (mesenteric, bronchial, and mediastinal lymph nodes) and other organs such as the lungs, liver, spleen, and kidney are indicative of the disease’s internal form [28, 29]. The disease manifested in cattle as cutaneous, visceral, and mastitis forms [10]. Unfortunately, internal abscesses are often undetected, whereas external abscesses are often responsible for clinical manifestations and disease transmission [5].

The gross pathological patterns observed in this study are consistent with previous reports describing caseous nodule formation, suppurative bronchopneumonia, calcification, and the classical “onion-skin” appearance of abscesses as pathognomonic features of CLA [1, 5, 11, 30, 31]. The onion-like lamellated arrangement of fibrous tissue results from sequential stages of necrosis and capsule formation within the abscess [32, 33]. Results were also consistent with earlier research [33, 34] that found similar lesions in experimental goats injected with *C. pseudotuberculosis*. The accumulation of caseous material in lymph nodes (including internal and mesenteric nodes) and visceral organs (lungs, liver, spleen, and kidneys) is a key characteristic of the internal form of CLA in small ruminants [31, 35, 36]. Karimi et al. [37] also reported the isolation of *C. pseudotuberculosis* from an unusual site in a goat’s spinal canal. In contrast, *C. pseudotuberculosis* infection is rare in cattle, although it was first identified as a cause of bovine lymphangitis in 1888.

The bacterium has also been associated with mastitis, either naturally [38] or experimentally [39], and infections in Israeli dairy cattle have presented with cutaneous, mastitis, and visceral manifestations [10]. Globally, there is limited information on CLA in cattle, and to date, no cases have been reported in Bangladesh. Although CLA in cattle mostly occurs in the superficial form and hardly any lesions are reported in the internal form [9], the pathology of rare cases of CLA (visceral type) in slaughtered cattle with exclusive involvement of mesenteric lymph nodes and lungs was identified in this investigation. Detection of internal CLA in cattle is epidemiologically important because infections frequently occur without visible external abscesses and remain undetected during ante-mortem examination. Identification of internal lesions in lymph nodes and visceral organs at slaughter provides critical evidence of subclinical infection and contributes to more accurate estimation of disease prevalence [1]. *C. pseudotuberculosis* can also produce lymph node lesions and liver abscesses in humans [8, 40, 41]. Information on CLA human case studies in Bangladesh is scarce.

Further, impression smear staining revealed pleomorphic, blue-colored, Gram-positive coccobacilli arranged in a characteristic Chinese-letter pattern within the mesenteric lymph nodes of animals, consistent with previous descriptions of *C. pseudotuberculosis* morphology [1, 9].

The histopathological findings observed in this study were consistent with previously described tissue responses to *C. pseudotuberculosis* infection [5, 12, 42, 43]. Caseous necrosis with varying degrees of mineralization reflects the chronic nature of CLA. The ability of bacteria to evade the immune system and their tendency to spread to multiple organs are responsible for the development of chronic lesions in CLA, including the formation of multiple abscesses and a lamellate layer. Its pathogenic potential is determined by virulence factors such as phospholipase D and mycolic acids [4], which help the bacteria evade a robust host immune response. These results were in line with earlier studies [1, 43]. The presence of Gram-positive coccobacilli arranged in a characteristic Chinese-letter pattern within necrotic foci provides pathognomonic histological confirmation of *C. pseudotuberculosis* infection [1]. The results revealed lesions mostly in the mesenteric lymph nodes of goats (28), cattle (3), and sheep (1), followed by the livers of goats (8) and sheep (1), then the lungs of goats (3) and cattle (1), the spleen of goats (3), and the kidney of a goat (1). *C. pseudotuberculosis* has a special affinity for lymphoid organs, and from lymphoid organs, bacteria disseminate to other visceral organs. Generally, the lymph node is the primary target replication site of *C. pseudotuberculosis*, and the liver and lungs are two of the main internal target organs in CLA [17].

After standardization of PCR protocols, segments of the *16S rRNA* gene of *C. pseudotuberculosis* were detected in goats, sheep, and cattle. The *16S rRNA* gene was also detected by other researchers by PCR [19, 21]. According to Cetinkaya et al. [17], *16S rRNA* gene-based molecular identification of *C. pseudotuberculosis* is favored because it has been shown to be a highly specific method for diagnosing CLA. However, as this gene cannot differentiate between *C. pseudotuberculosis* and *C. ulcerans*, duplex PCR targeting the pld and *rpoB* genes was used to provide a more specific and sensitive test. Previously, the *pld* and *rpoB* genes of *C. pseudotuberculosis* were effectively detected by PCR [19, 20]. Although bacterial culture remains a conventional diagnostic method, its usefulness is limited by the slow growth of *C. pseudotuberculosis*, contamination of necrotic tissues, and reduced bacterial viability in chronic or calcified lesions [4, 16, 44]. Nowadays, PCR techniques have been developed based on *16S rRNA* [17], *pld* [19], and *rpoB* [20] genes, offering high sensitivity, reproducibility, and diagnostic efficiency. In this study, we primarily focused on diagnosis through investigation of pathological features and on molecular detection by PCR. Serological tests are also recommended to detect antibodies to *C. pseudotuberculosis* at the herd level [14], but were not used in this study.

Our study detected CLA in 29.41% (30/102) of goats, 6.25% (1/16) of sheep, and 18% (9/50) of cattle. We did not conduct any statistical analysis due to sample limitations to determine species-specific variation in CLA occurrence in Bangladesh. The occurrence of CLA was apparently higher in goats than in cattle and sheep. This finding is consistent with previous reports indicating that CLA is more prevalent in goats and sheep than in cattle [9]. In this study, the occurrence of CLA in sheep was low, possibly due to smaller sample sizes. In the Mymensingh and Kushtia districts of Bangladesh, the seroprevalence of CLA in goats was 6.59% and 0.74%, respectively [13, 14]. The latter was much lower than in the present study. This may be because the serological test could be less sensitive than PCR detection of microbial genes [18, 45]. However, worldwide, seroprevalence varies from 35–95% [18]. In neighboring country India, sporadic reports indicate a CLA prevalence of 4.7% based on clinical examination and 2.4% confirmed by culture and PCR, with occasional outbreaks suggesting endemic transmission [46]. Additionally, in the Andaman and Nicobar Islands of India, CLA was first confirmed in goats with an attack rate of 12.02% [47]. In Pakistan, an abattoir-based study found that within-flock CLA prevalence in sheep ranged from 0 to 33.3% based on clinical lymph node examination [48]. *C. pseudotuberculosis* was also detected in 19 of 25 examined Chinkara deer (*Gazella bennettii*), demonstrating a high regional prevalence and suggesting potential wildlife involvement in disease ecology [49] in Pakistan. In the Somal region of Dohuk Province, Iraq, Issa et al. [50] reported a CLA prevalence of 1.9% in the slaughtered sheep. In the Beni-Suef Governorate of Egypt, CLA was identified in 13.9% of sheep and 8.6% of goats from March 2016 to April 2017 [51]. In Algeria, the molecular prevalence of CLA was slightly higher in sheep (8.9%) than in goats (1.6%); this may be because sheep are a common small ruminant species in Algeria, whereas goats are rarely reared [52]. On the other hand, the occurrence of CLA in cattle is limited worldwide. At the Bishoftu municipal abattoir in Ethiopia, a single sporadic case of CLA in cattle involving the pre-scapular lymph node was found in a cross-sectional abattoir study [42]. An outbreak of CLA occurred in southern Portugal, affecting 11% of dairy cattle, involving lymph nodes and internal organs [38]. Sood et al. [9] reported an extremely rare case of mesenteric lymphadenitis in a cow calf in India.

Our study indicates that CLA exists in small ruminants and cattle in Bangladesh. Molecular confirmation of *C. pseudotuberculosis* helps distinguish CLA from other granulomatous conditions, including bovine tuberculosis, and underscores the potential role of cattle as asymptomatic carriers that contribute to interspecies transmission in mixed livestock systems [10]. The presence of CLA in cattle suggests that the disease can be easily transmitted from small ruminants to cattle, as previously described [10]. Transmission of *C. pseudotuberculosis* between species can result from close contact between small ruminants and cattle, mechanical transmission by arthropod vectors, and the organism’s capacity to survive for prolonged periods in contaminated habitats [9, 38]. As CLA also has zoonotic importance, effective detection, management of infected slaughtered animals, and prevention of animal-to-animal transmission are essential to protect public health and animal welfare.

## 5. Conclusions

To the authors’ knowledge, this is the first report detecting internal CLA in slaughtered goats, sheep, and cattle in Bangladesh using pathological and molecular detection PCR. The concordance between gross pathology, histopathology, and molecular detection demonstrates the reliability of these methods for slaughterhouse-based surveillance. Enhancing routine post-mortem inspection, in combination with molecular detection, would improve early detection, facilitate effective disease control, and reduce economic losses related to CLA. These results also have significant public health implications due to *C. pseudotuberculosis*’s zoonotic potential, especially for individuals occupationally exposed to livestock and slaughterhouse environments. Further research is recommended on large-scale epidemiological surveys, molecular strain typing, and vaccine development to support effective prevention and control strategies for CLA in Bangladesh.

## Data Availability

The data presented in this study are available from the corresponding author upon reasonable request.

## List of abbreviations

CLA, caseous lymphadenitis; DNA, deoxyribonucleic acid; °C, degree Celsius; µl, microliter; min, minutes; pmol, picomole; PCR, polymerase chain reaction; PLD, phospholipase; D; *rpoB*, RNA polymerase subunit beta; *16S rRNA*, 16 subunit ribosomal ribonucleic acid; sec, seconds.
